# Manifestation von Morphea bei einer Patientin mit Myasthenia gravis unter Therapie mit Zilucoplan

**DOI:** 10.1111/ddg.15919_g

**Published:** 2026-02-05

**Authors:** Phoebe Wellmann, Jana Zschüntzsch, Michael P. Schön, Rotraut Mössner

**Affiliations:** ^1^ Klinik für Dermatologie Venerologie und Allergologie Universitätsmedizin Göttingen; ^2^ Klinik für Neurologie Universitätsmedizin Göttingen

Sehr geehrte Herausgeber,

Zilucoplan, ein subkutan verabreichter Inhibitor des Komplementfaktors C5, wurde im Dezember 2023 von der *Europäischen Kommission* zur Behandlung der Anti‐Acetylcholinrezeptor‐Antikörper‐positiven Myasthenia gravis (MG) zugelassen.^1^ Es handelt sich um ein synthetisches, makrozyklisches Peptid aus 15 Aminosäuren.^2^ In klinischen Studien wurde Zilucoplan im Allgemeinen gut vertragen. Zu den häufigsten Nebenwirkungen zählten Reaktionen an der Injektionsstelle, Infektionen der oberen Atemwege, Durchfall und Morphea.^3^, ^4^

Eine 36‐jährige Frau zeigte eine unzureichende Kontrolle der MG‐Symptomatik unter systemischer Kortikosteroidtherapie und wiederholten Zyklen mit intravenösen Immunglobulinen. Vorangegangene Therapien mit Methylprednisolon, Azathioprin, Mycophenolatmofetil und Methotrexat mussten aufgrund unzureichender klinischer Wirksamkeit oder Nebenwirkungen abgebrochen werden. Die Krankheitsschwere nach der Myasthenia‐Gravis‐Foundation‐of‐America (MGFA)‐Klassifikation betrug IIIa. Sie nahm an der RAISE‐Studie (MG0010)^3^ und deren Verlängerung RAISE‐XT (MG0011)^5^ teil, in denen Zilucoplan zur Behandlung der MG untersucht wurde (September 2021 bis März 2024). Nach Abschluss der Studien wurde die Behandlung mit kommerziellem Zilucoplan fortgesetzt. Nach 2,5 Jahren wirksamer Behandlung mit Zilucoplan, gemessen an einer Verbesserung des MG‐ADL (Aktivitäten des täglichen Lebens) von 10 Punkten zu Beginn auf 0 Punkte, stellte sich die Patientin mit hyperpigmentierten und sklerotischen Plaques an den lateralen Brustbereichen, submammär, in den Leistenregionen sowie mit einer 4–5 cm großen sklerotischen Makula an der linken Schulter vor (Abbildung 1). Diese Hautveränderungen bestanden seit etwa einem Jahr. Zusätzlich berichtete sie über gelegentliche Gliederschmerzen sowie episodische kälteinduzierte bläuliche Verfärbungen, Steifigkeit und Juckreiz der Finger.

Der histologische Befund einer Hautbiopsie von der linken Schulter zeigte sich passend zu einer Morphea: milde basale Hyperpigmentierung, kompakte Orthokeratose, homogenisiertes kollagenes Gewebe in der tiefen Dermis und milde perivaskuläre lymphozytäre Infiltrate mit gelegentlichen Plasmazellen. In der Autoimmundiagnostik zeigten sich positive ANA‐Titer (granuläres Nukleoplasma 1:1000, Zytoplasma‐positiv 1:320), erhöhte SSA‐Antikörper > 240,0 U/ml sowie positive Pm‐Scl‐75‐Antikörper. Für die systemische Sklerodermie typische Antikörper, darunter SCL‐70, CENP‐B, RNA‐Polymerase‐III und Fibrillarin, waren negativ. Auf Nachfrage wurde Mundtrockenheit angegeben. Eine Unterlippenbiopsie war unauffällig, eine Kapillarmikroskopie der Nagelfalz zeigte unspezifische Veränderungen. Es wurde eine Morphea vom Plaque‐Typ diagnostiziert. Die Hautveränderungen wurden mit hochpotenten Glukokortikoiden und UVA1‐Phototherapie behandelt. Die Phototherapie wurde nach 9 Wochen wegen fehlender Besserung abgebrochen, während die topische Glukokortikoidtherapie fortgeführt wurde. Eine systemische Therapie mit Methotrexat, wie sie in aktuellen Leitlinien als Erstlinientherapie bei Morphea empfohlen wird, wurde nicht erneut eingeleitet, da die Patientin bereits eine dreimonatige Therapie mit Methotrexat wegen MG erhalten hatte, die aufgrund von Nebenwirkungen (unter anderem Übelkeit, Erbrechen, Schmerzen und grippeähnliche Symptome) abgebrochen werden musste.^6^ Trotz der Manifestation von Morphea entschied sich die Patientin zunächst, die Therapie mit Zilucoplan aufgrund der ausgezeichneten Besserung der MG‐Symptome fortzusetzen.

Morphea, auch als zirkumskripte Sklerodermie bezeichnet, ist eine seltene entzündliche Bindegewebserkrankung, die primär die Haut betrifft, aber auch Faszien, Muskeln, Knochen und das zentrale Nervensystem betreffen kann. Die Morphea vom Plaque‐Typ, die häufigste Unterform, ist charakterisiert durch erythematöse, sklerotische Läsionen mit einem lividen Rand im aktiven Stadium. Im Verlauf kommt es zu zentraler Sklerose und potenziell atrophen Arealen mit Hypo‐ oder Hyperpigmentierung.^6^, ^7^ Behandlungsoptionen umfassen topische Therapien, UV‐Therapien, systemische Therapien (zum Beispiel Methotrexat, Glukokortikoide) und Physiotherapie.^6^ Mögliche Auslöser von Morphea sind Infektionen, mechanische Traumata, eine Strahlentherapie und Medikamente wie Bleomycin, D‐Penicillamin, Vitamin K1, L‐5‐Hydroxytryptophan mit Carbidopa sowie Balicatib.^8^ Laut Fachinformation ist Morphea auch eine häufige Nebenwirkung von Zilucoplan.^4^ Sie wurde als unerwünschte Arzneimittelwirkung in der offenen Langzeit‐Verlängerungsstudie MG0011 beobachtet. An dieser Studie nahmen 34 Patienten (17%) aus der vorangegangenen doppelt verblindete Phase‐II‐Studie MG0009 und 166 Patienten (83%) aus der Phase‐III‐Studie MG0010 teil. Morphea war in der von Howard et al. durchgeführten Zwischenanalyse der RAISE‐XT‐Studie als Nebenwirkung nicht beschrieben, wobei der Stichtag für die Datenerhebung im September 2022 lag. Zu diesem Zeitpunkt betrug die mediane Exposition gegenüber Zilucoplan 1,2 Jahre, bei einer kumulativen Exposition von 321,4 Patientenjahren.^5^ Morphea‐Fälle wurden erst nach diesem Stichtag festgestellt, als weitere Daten erhoben wurden. Die meisten Morphea‐Fälle traten nach mehr als einem Jahr unter Zilucoplan auf, waren leicht bis moderat ausgeprägt und erforderten keinen Therapieabbruch.^4^ Bei unserer Patientin wurde Zilucoplan nach 32 Monaten abgesetzt, da sich die Hautveränderungen trotz fortgesetzter topischer Glukokortikoidtherapie langsam verschlechterten. Nach Absetzen von Zilucoplan und Fortsetzung der topischen Behandlung blieb die Morphea unverändert. Die MG verschlechterte sich jedoch, sodass eine Therapie mit einem Inhibitor des neonatalen Fc‐Rezeptors begonnen wurde.

Der Pathomechanismus der Morphea ist nicht vollständig geklärt. Sie wird als Autoimmunerkrankung eingestuft, die initial durch eine T‐Zell‐vermittelte periadnexielle sowie perivaskuläre Entzündung der Haut gekennzeichnet ist. In frühen Stadien kommt es zur Rekrutierung von proinflammatorischen und fibroblastenaktivierenden TH1‐ und TH17‐Zellen, im Verlauf zu einer Verschiebung hin zu TH2‐Zytokinen und vermehrter Fibrose.^6^ Venneker et al. vermuten, dass eine verminderte Expression von komplementregulatorischen Molekülen im Endothel zur Gefäßschädigung und Fibrose beitragen kann.^9^

Auch wenn eine zufällige, unabhängige Manifestation von Morphea nicht ausgeschlossen werden kann, wird Morphea in der Fachinformation von Zilucoplan als häufige Nebenwirkung aufgeführt – definiert als Auftreten bei > 1/100 und < 1/10 der Behandelten.^4^ Demgegenüber liegt die Inzidenz von Morphea in der Allgemeinbevölkerung bei 4 bis 27 pro einer Million Personen.^6^, ^7^ Es gibt nur begrenzte Daten zur Inzidenz von Morphea bei MG‐Patienten, jedoch scheint sie bei Autoimmunerkrankungen insgesamt höher zu sein als in der Allgemeinbevölkerung.^10^

Nach unserem Wissen wurde Morphea bisher nicht als Nebenwirkung anderer C5‐Komplementinhibitoren beschrieben. Es bleibt zu untersuchen, ob es sich um einen substanzspezifischen oder einen pharmakologisch bedingten Off‐Target‐Effekt handelt, wobei es bisher keine Hinweise auf einen Klasseneffekt gibt. Weitere Studien sind notwendig, um den Pathomechanismus von durch Zilucoplan induzierter Morphea zu klären und das Management zu optimieren.

## DANKSAGUNG

Open access Veröffentlichung ermöglicht und organisiert durch Projekt DEAL.

## INTERESSENKONFLIKT

P.W. meldet keinen Interessenkonflikt. J.Z. war Berater und/oder erhielt Vortragshonorare und/oder erhielt Zuschüsse und/oder nahm an klinischen Studien der folgenden Unternehmen teil: Alexion, Amicus, Argenx, Kedrion, Roche, Sanofi, UCB. M.P.S. war Berater und/oder erhielt Vortragshonorare und/oder erhielt Zuschüsse und/oder nahm an klinischen Studien der folgenden Unternehmen teil: AbbVie, Almirall, Biogen, Boehringer‐Ingelheim, Janssen‐Cilag, Leo, Lilly, Novartis, UCB. R.M. war Berater und/oder erhielt Vortragshonorare und/oder erhielt Zuschüsse und/oder nahm an klinischen Studien der folgenden Unternehmen teil: AbbVie, Almirall, Biogen, Boehringer‐Ingelheim, Celgene, Janssen‐Cilag, Leo Pharma, Lilly, MSD Sharp & Dohme, Novartis, Pfizer, UCB.

**ABBILDUNG 1:**
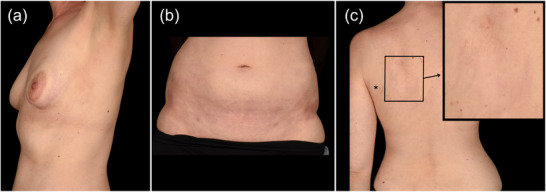
Klinische Bilder von Morphea bei der Erstvorstellung. (a) Hyperpigmentierte, sklerotische Makula, halbkreisförmig, auf der linken lateralen Seite der Brust mit deutlichem periareolärem Rand. (b) Hyperpigmentierte, sklerotische Makula in der Inguinalregion. (c) 4–5 cm große sklerotische Makula an der linken Schulter. Das Sternchen kennzeichnet eine Narbe von einer Exzision vor Jahren.
